# Economic burden of hypoglycemia for type II diabetes mellitus patients in Malaysia

**DOI:** 10.1371/journal.pone.0211248

**Published:** 2019-10-25

**Authors:** Syed Mohamed Aljunid, Yin Nwe Aung, Aniza Ismail, Siti Athirah Zafirah Abdul Rashid, Amrizal M. Nur, Julius Cheah, Priya Matzen

**Affiliations:** 1 International Centre for Casemix and Clinical Coding, Faculty of Medicine, Universiti Kebangsaan Malaysia, Kuala Lumpur, Malaysia; 2 Department of Health Policy and Management, Faculty of Public Health, Kuwait University, Hawalli, Kuwait; 3 Department of Pathology and Community Medicine, Faculty of Medicine and Health Sciences, UCSI University, Kuala Lumpur, Malaysia; 4 Market Access and Public Affairs, Novo Nordisk Pharma (Malaysia) Sdn Bhd, Kuala Lumpur, Malaysia; 5 Global Public Health, Jeffrey Cheah School of Medicine and Health Sciences, Monash University Malaysia, Kuala Lumpur, Malaysia; 6 Human Development and Health, Faculty of Medicine, University of Southampton, Southampton, United Kingdom; University of Bologna, ITALY

## Abstract

This study mainly aims to identify the direct cost and economic burden of hypoglycemia for patients with type II diabetes mellitus in Malaysia. A cross-sectional study explored the cost incurred for hypoglycemia among patients admitted to University Kebangsaan Malaysia Medical Centre (UKMMC). The study covered patients aged 20–79 years hospitalized with a primary diagnosis of ICD-10 hypoglycemia and discharged between January 2010 and September 2015 according to the casemix database. A costing analysis was done through a step-down approach from the perspective of health providers. Cost data were collected for three levels of cost centers with the help of a hospital-costing template. The costing data from UKMMC were used to estimate the national burden of hypoglycemia among type II diabetics for the whole country. Of 244 diabetes patients admitted primarily for hypoglycemia to UKMMC, 52% were female and 88% were over 50 years old. The cost increased with severity. Managing a hypoglycemic case requires five days (median) of inpatient stay on average, with a range of 2–26 days, and costs RM 8,949 (USD 2,289). Of the total cost, 30% related to ward (final cost center), 16% to ICU, and 15% to pharmacy (secondary-level cost center) services. Considering that 3.2% of all admissions were hypoglycemia related, the total annual cost of hypoglycemia care for adult diabetics in Malaysia is estimated at RM 117.4 (USD 30.0) million, which translates to 0.5% of the Ministry of Health budget. Hypoglycemia imposes a substantial economic impact even without the direct and indirect cost incurred by patients and other cost of complications. Diabetic management needs to include proper diabetic care and health education to reduce episodes of hypoglycemia.

## Introduction

Hypoglycemia is not an uncommon presentation at accident and emergency (A&E) departments. An estimated 2–4% of diabetes deaths are due to hypoglycemia [1]. Hypoglycemic episodes are inevitable in 90% of diabetics with insulin therapy [2]; those on certain anti-hyperglycemic medicine are also at risk. The elderly and those with comorbid conditions are more likely to have severe hypoglycemia [3]. The incidence of hypoglycemia varies with studies. On average, type 1 diabetics had two symptomatic hypoglycemia episodes per week [4] and one severe hypoglycemia episode per year [5], with a lesser occurrence among type II diabetes patients [2]. However, type II diabetics with progressive insulin deficiency, long duration of disease, and tight glycemic control also have similar risk of hypoglycemia as type 1 diabetics [5].

Of type II diabetic patients with different anti-diabetic regimens, 11% suffered one or more hypoglycemic episodes per year. Miller et al. reported that 24.5% of patients reported at least one hypoglycemic episode in 3 months [6, 7]. Mild hypoglycemic attacks occur frequently, as often as 1–2 episodes per week [8] and severe hypoglycemic attacks occur less frequently with an incidence rate of 1 to 2.7 episodes per patient per year [8, 9].

Diabetes mellitus (DM) is one of the commonest chronic non-communicable diseases globally. Its incidence and prevalence are escalating, and the Asia-Pacific region is at the forefront of the current epidemic. It is estimated that 8.8% of the adult population in Southeast Asia region had diabetes as of 2015 [10]. DM prevalence among Malaysians of ages 20–79 is estimated to be 17.5% [11], ranking third in the Asia-Pacific region [12, 13]. Malaysia is estimated to have 2.48 million diabetics in 2030 compared to 0.94 million in 2000, which translates to an increase of 164% [14].

The chronic nature of diabetes and its devastating complications make it a very costly disease. To prevent the risk of acute and chronic complications, diabetic patients require continuous medical care [2, 15]. Hypoglycemia has unpredictable and undesirable side effects among diabetic patients. Frequent and potentially fatal complications may occur among hypoglycemic patients with type 1 or type II diabetes treated with insulin, and in patients with type II diabetes treated with certain oral anti-diabetic medicines.

Hypoglycemic attacks require defense mechanism against falling serum glucose, and frequent attacks result in increasing cycles of recurrent hypoglycemia [13]. Fear of hypoglycemia is not uncommon in patients with diabetes. Experiences of severe hypoglycemic episodes increase the fear of future hypoglycemic events. Approximately 40% of patients admitted that fear of hypoglycemia caused them to maintain their blood glucose levels at higher than recommended values [14]. This questions the compliance of anti-diabetic medicine with possible complications due to uncontrolled diabetes and increased health-care costs. Although hypoglycemia causes a significant burden, its cost in Malaysia is yet unknown. This study identifies the direct cost and economic burden of hypoglycemia for patients with type II diabetes mellitus on insulin in Malaysia.

## Methods

A cross-sectional study was conducted to determine the cost of hypoglycemia among patients admitted to the Universiti Kebangsaan Malaysia Medical Centre (UKMMC). Patients discharged from January 2010 to September 2015, based on UKMMC’s electronic medical database, were classified into diagnosis-related groups (DRG) with the MY-DRG® grouping software. Those aged 20–79 years with hypoglycemia as a primary reason for admission according to the casemix database were identified from ICD 10 codes. The ICD 10 codes associated with hypoglycemia, namely, E16.0 (drug-induced hypoglycemia without coma, E16.1 (other hypoglycemia), and E16.2 (hypoglycemia unspecified), were included in the study.

The study was approved by the Research and Ethics Committee of Universiti Kebangsaan Malaysia (Approval Code:FF-2016-147). The study does not require informed consent because the data was extracted from Universiti Kebangsaan Malaysia Medical Centre (UKMMC) MY-DRG database and did not involve interviews of patients.

UKMMC is a 1,000-bed public teaching hospital owned by Ministry of Education. Patients managed in this hospital are heavily subsidized by the government and most of them paid out-of-pocket charges of RM 30 (USD 7.67) for outpatient services and RM 50 (USD12.80) per day for inpatient care.

A costing analysis based on the step-down approach was carried out. We followed the guidelines developed by Shepard et al. [16] for the hospital costing analysis. Finance staffs in the hospital were given a costing template to retrieve financial data from the hospital records. The template classifies the departments into three levels of cost centers: overhead cost centers (e.g., administration, utilities, maintenance), intermediate cost centers (e.g., pharmacy, radiology) and final cost centers (all wards and all clinics). Our costing template included 17 overhead cost centers, 16 intermediate cost centers, and 25 final cost centers. Information on financial expenditures and output for each cost center was recorded. The financial expenditure for each cost center covers the cost of human resource, materials, buildings, and equipment. The information recoded includes the total expenditure, total number discharges, inpatient days, number of patient visits for outpatient clinics, and floor space. All the information on activities reflecting the workload such as number of discharges, inpatient days, floor space, and number of outpatient visits were gathered for appropriate cost allocation. The useful life of building was assumed to be 20 years, while the useful life of equipment was assumed to be 5 years. We applied a 5% discount rate to impute depreciation cost of capital outlay.

Both capital cost (building, equipment, and furniture cost) and recurrent cost (staff salary and other recurrent cost) were combined in estimating the cost for each cost center. The final allocated costs for each inpatient cost center were then divided by the total units of inpatient days to obtain the cost of providing services on a per-patient per-day of stay basis, referred to as unit cost. The unit cost is finally multiplied with the individual patient’s length of stay to obtain the cost of care per patient per discharge. All these steps were simplified by using the Clinical Cost Modelling Software Version 2.1 (CCM Ver. 2.1). CCM is the step-down costing tool used by the casemix system in UKMMC.

The cost is triangulated by developing clinical pathways for a hypoglycemic episode. An expert group meeting was held to develop the common clinical pathways of managing hypoglycemia. These experts include physicians, endocrinologists, and pharmacists working in the A&E department and in the clinical departments of the hospital.

In imputing the national burden, estimates for incidence and prevalence of diabetes were obtained from NMHS 2015 [8] and the International Diabetes Federation [9]. The possible episode of hypoglycemia incidence was estimated from the Malaysian Hypoglycaemia Assessment Tool Study (HAT study) [17]. The unit cost for management of hypoglycemia calculated in the step-down approach was used to estimate the burden. Sensitivity analysis was carried out by varying the incidence of hypoglycemia to obtain the worst-case scenario and best-case scenario.

## Results

As shown in Table 1, 903 cases (0.54% of the total cases) discharged from UKMMC between January 2010 and September 2015 had a diagnosis of hypoglycemia.

**Table 1:** Number of hypoglycemia patients by year in the UKMMC casemix database.

Year	Nos.	Total Discharges	Hypoglycemia Discharges as Percentage of Total
2010	199	32,144	0.62%
2011	157	26,262	0.60%
2012	180	28,728	0.63%
2013	177	33,473	0.53%
2014	119	27,303	0.44%
2015	71	18,623	0.38%
**Total**	903	166,533	0.54%

These cases were classified into the endocrine, Nutrition, and metabolism (33.4%), respiratory system (12.6%), and cardiovascular system (9.7%) MY-DRG® casemix main groups. The central nervous and nephro-urinary systems contributed 6.4% of the hypoglycemia cases (Table 2).

**Table 2:** Hypoglycemia by casemix main group (CMG).

No	Casemix Main Groups (CMG)	Frequency	Percentage
1	Endocrine system, nutrition & metabolism groups	302	33.4
2	Respiratory system groups	114	12.6
3	Cardiovascular system groups	88	9.7
4	Central nervous system groups	58	6.4
5	Nephro-urinary system groups	58	6.4
6	Infectious & parasitic diseases groups	51	5.6
7	Musculoskeletal system & connective tissue groups	47	5.2
8	Digestive system groups	45	5
9	Hepatobiliary & pancreatic system groups	38	4.2
10	Female reproductive system groups	26	2.9
11	Skin, subcutaneous tissue & breast groups	23	2.5
12	Hemopoeitic & immune system groups	17	1.9
13	Ear, nose, mouth, & throat groups	10	1.1
14	Myeloproliferative system & neoplasms groups	7	0.8
15	Mental health and behavioral groups	5	0.6
16	Injuries, poisonings & toxic effects of drugs groups	4	0.4
17	Eye and adnexa groups	3	0.3
18	Male reproductive system groups	2	0.2
19	Deliveries groups	2	0.2
20	Factors influencing health status & other contacts with health services groups	2	0.2
21	Substance abuse & dependence groups	1	0.1
	Total	903	100

Most of the hypoglycemic patients were admitted to the medical ward (91.7%), and 8.1% were admitted to the surgical unit.

Of the 302 hypoglycemic cases with endocrine problems, diabetes in particular, 244 cases were admitted primarily for hypoglycemia and 58 were admitted for hypoglycemia as a secondary reason (Table 3).

**Table 3:** Type of hypoglycemia in the CMG endocrine system, nutrition & metabolism.

Type of Hypoglycemia	Frequency	Percentage
Hypoglicemia as main diagnosis	244	80.8%
Hypoglicemia as secondary diagnosis	58	19.2%
Total	302	100

Only 244 cases with hypoglycemia as primary reason were included in the study for further analysis. Females were more likely to suffer from hypoglycemia, as almost 52% of the cases were female. About 99.2% of all cases were discharged well. The MY-DRG® system classifies patients into one of three levels of severity. Cases of severity level 1 do not have complications or comorbidities, whereas severity level 2 implies minor complications and comorbidities. Patients in severity level 3 have major complications and comorbidities. About 57.4% of the primary hypoglycemia cases were in severity level 3. Most (88.1%) of the patients suffering from hypoglycemic attacks were in the age group of 50–79 years, and 55.3% of the cases required not more than 5 days of hospital admission. The median length of stay is 5 days, and the IQR is 4 (4–8) days (Table 4).

**Table 4:** Characteristics of hypoglycemia in the CMG endocrine system, nutrition and metabolism.

Characteristics		Hypoglycemia as primary diagnosis (n = 244)
Gender (Female, %)		127 (52%)
Discharged status	Discharged to home	242 (99.2%)
	Others	2 (0.8%)
Severity level	Severity level 1	23 (9.4%)
	Severity level 2	81(33.2%)
	Severity level 3	140 (57.4%)
Age group	20–29 years	3 (1.2%)
	30–39 years	8 (3.3%)
	40–49 years	18 (7.4%)
	50–59 years	34 (13.9%)
	60–69 years	84 (34.4%)
	70–79 years	97 (39.8%)
Length of stay	< = 5 day	135 (55.3%)
	6–10 days	77 (31.6%)
	11–15 day	18 (7.4%)
	16–20 day	10 (4.1%)
	21–25 day	3 (1.2%)
	26–30 days	1 (0.4%)

The treatment cost for a hypoglycemia patient at the UKMMC A&E department is RM 741 (USD 190) per visit. Generally, the cost of hypoglycemic cases admitted to the medical unit is lower than in other units: RM 1,375 (USD 352) per day at the medical unit compared to RM 1,679 (USD 430) per day at the obstetrics and gynecology unit, and (RM 2,611 (USD 668) per day at the surgical unit. Out of 903 cases diagnosed with hypoglycemia, 828 were from the medical ward, 73 from the surgical ward, and only 2 from the O&G unit.

The average cost of hypoglycemia care varies with the severity level. Although the mean cost at severity 1 and 2 is RM7,054 (USD 1,804) and RM7,333 (USD 1,875), respectively, it reaches RM10,195 (USD 2,607) at severity level 3.

The mean cost of hypoglycemia is slightly lower among females than males, but the difference is not statistically significant. Age has a significant influence on the cost of hypoglycemia care. Hypoglycemia patients in the 40–49 age group have a higher mean cost, at RM 11,000 (USD 2,813), than those in the 20–39 and 60–79 age groups. The median cost was RM 6,875 (USD 1,758) for a primary diagnosis and RM 11,000 (USD 2,813) for a secondary diagnosis of hypoglycemia. The median length of stay was 5 days for a primary diagnosis and 8 days for a secondary diagnosis. This study focused on diabetic cases with hypoglycemia as the primary reason for admission, where the median cost was RM 6,875 (USD 1,758) with a median hospital stay of 5 days (Table 5).

**Table 5:** The cost (RM) of hypoglycemia by severity level, gender, and age.

		N	Mean (RM)	SD (RM)	Med (RM)	Min (RM)	Max (RM)	IQR (RM)
Severity level ^a^	Severity level 1	23	7,054	3,807	5,500	2,750	15,125	4,125
	Severity level 2	81	7,333	4,165	6,875	2,750	26,125	5,500
	Severity level 3	140	10,195	7,065	8,250	2,750	35,750	5,500
Gender ^b^	Male	117	9,084	6,719	6,875	2,750	3,570	4,125
	Female	127	8,824	5,574	6,875	2,750	3,570	5,500
Age group ^c^	20–39	11	8,375	4,579	6,875	4,125	17,875	6,875
	40–59	52	11,000	8,110	8,250	2,750	35,750	6,875
	60–79	181	8,394	5,434	6,875	2,750	31,625	4,125
	All Cases	244	8,949	6,138	6,875	2,750	35,750	5,500

^a^ Anova: F = 7.13, p = 0.0010

^b^ t-test: t = 0.3307, p = 0.7412

^c^ Anova: F = 3.77, p = 0.0234

The cost incurred at each cost centers was determined. Of the total cost, 30% was attributed to the final cost center (the ward services cost), 16% to ICU services, and 15% to pharmacy and drug services.

The incidence and prevalence assumptions were based on information from the International Diabetes Federation [8], the HAT study [17], and NMHS 2015 [9]. According to NMHS 2015, 17.5% of adult Malaysians are estimated to have diabetes mellitus, of whom 8.3% represent known cases. Of the known diabetics, 25.1% are on insulin therapy, which is equivalent to 404,619 people. According to a conservative estimate by HAT, the annual incidence among Type II DM patients is 47.1% for any type of hypoglycemic episode and 16.8% for severe hypoglycemia [17]. We developed a base-case, a best-case, and a worst-case scenario assuming the number of cases requiring hospital admission and the mean cost of care in UKMMC with hypoglycemia as the main diagnosis for severity levels 1 to 3. We estimated that 5.9% of severe hypoglycemia cases (23,872 patients) require hospital admission in the worst-case scenario based on the retrospective arm findings of the HAT study. The base-case scenario is based on an expert group discussion with local clinicians, who reached a consensus on hypoglycemia-related hospital admissions of 3.2% (12,948 patients). The best-case-scenario assumed 2.5% prevalence (10,115 patients) based on the prospective arm findings of the HAT study on hospital admissions for hypoglycemia. We assume that all these cases require hospital admission at least once.

The total annual cost of care for hypoglycemia among adult diabetes patients in Malaysia was estimated at RM 117.4 (USD 30.0) million, approximately 0.5% of the Ministry of Health annual budget allocation of RM 22.16 (USD 5.67) billion in 2014 [18]. The national economic burden estimates based on the best-case scenario and the worst-case scenario range from RM 91.7 (USD 23.5) million to RM 216.5 (USD 55.4) million (Table 6)

**Table 6:** The estimated cost and economic burden of hypoglycemia.

Hypoglycemia as primary diagnosis	% of cases	Mean cost per admission (RM)	Worst-case scenario (5.9% admission)	Base-scenario (3.2% admission)	Best-case scenario (2.5% admission)
Severity level 1	9.4	7,054	15,829,227	8,585,371	6,707,321
Severity level 2	33.2	7,333	58,118,921	31,522,127	24,626,662
Severity level 3	57.4	10,195	142,522,937	77,300,576	60,391,075
Total cost		8,949	216,471,136	**117,408,074**	91,725,057
% MOH budget (RM 22,160,380,300)			1.0	**0.5**	0.4

## Discussion

Proper glycemic control is required to minimize the risk of microvascular complications of diabetes. At the same time, diabetes management commonly results in hypoglycemic episodes. The occurrence of hypoglycemia is widely variable. The frequency of severe hypoglycemia requiring emergency services in patients receiving insulin therapy depends on how diabetes is managed for type 1 and type II DM. Incidence rates were 11.5 and 11.8 events per 100 patient-years for type 1 and type II patients treated with insulin, respectively [18]. A study in Canada stated that 1.9% of individuals had at least one hypoglycemia-related A&E visits, and 0.1% were admitted to hospital. In terms of the incidence rate, 5.2 cases and 0.3 cases per 1000 patient-years required A&E visits and hospital admission, respectively [19]. In the HAT study, hypoglycemic episodes of hospital admissions among DM cases in Malaysia were measured both prospectively and retrospectively. The prospective and retrospective findings of the HAT study were reviewed, and the most conservative estimates were selected and subsequently validated by specialists from different hospitals in Malaysia. Of the type II DM cases with insulin, 47.1% experienced some type of hypoglycemia and16.8% had severe hypoglycemic episodes within a 6-month period [17]. Based on their clinical experience, the expert group of specialists determined a prevalence rate of 3.2% for hypoglycemia requiring at least one hospital admission. The incidence of hospital admission was conservatively estimated as 3.2 episodes per person-year compared to 10 episodes per person-year for the worst-case scenario. Even with the best-case scenario, the incidence of admission due to hypoglycemic episodes is significantly higher than elsewhere [19, 20].

Several investigations are required to confirm and manage hypoglycemia cases appropriately. The duration of hospital stay may vary from place to place according to severity and the management protocol. The cost of hospital care is also dependent on how long the patient was admitted to the hospital. The mean length of hospital stay ranged from 5.5 to 9.8 days in most studies [21–23]. The median length of stay is 4 days in the Canada [20]. The findings from our study also provide similar estimates, with the mean length of hospital stay being 6.5 days and the median length 5 days. Due to the wide variation and huge standard deviation, the median length of stay was selected as the common length of hospital stay in this study.

Depending on the level of severity, a hypoglycemic condition may be treated at home, at the A&E department, or at hospital wards. The cost of care also varies with the level of severity. Our study showed that the cost of care at the A&E department was RM 741 (USD 190) per case. On the other hand, the mean cost of care for a patient admitted for hypoglycemia is RM 8,949 (USD 2,289) per admission.

The HAT study presented the proportion of patients admitted to hospital, but not the patients requiring an A&E visit prior to hospital admission. Other country studies stated that the proportion of hypoglycemia-related hospital admissions after the treatment in emergency departments (ED) varies between 11% and 28% [19, 24]. We do not have local estimates for this information, but based on our expert group review, it could be as high as 50%. However, we assumed that 5.9% of the cases require hospital admission in the worst-case scenario and 2.5% in the best-case-scenario from the findings of the Malaysian HAT study [17].

Although hypoglycemia-related hospitalization seems low, it can result in both clinical and economic burden considering the actual number of hypoglycemic episodes requiring hospital care. In this study, we have focused more on the cost of care for severe episodes of hypoglycemia at tertiary academic institutions and the total economic burden from hypoglycemia. The economic cost of hypoglycemia is difficult to compare because of differences in health care systems and in the definitions of hypoglycemia itself. Costing studies from the literature show that hypoglycemia admissions in Scotland cost USD 303 (£218) per person per day [23] compared to an average cost of USD 7,000 in Canada for a 7-day hospitalization due to hypoglycemia [20]. In Thailand, a patient with hypoglycemia requires 6 days of hospital stay on average, requiring almost USD 700 (THB 22,000) per episode [25]. A recent study in Korea estimated that the medical costs for a hypoglycemic event ranged from USD 17.28 to USD 1,857 at secondary and tertiary hospitals [26].

Although the individual cost of care in Malaysia is not significantly high compared to other countries, the number of episodes requiring health-care services at the hospital, and therefore the total cost of care, is considerably higher. Hypoglycemia care for type II DM patients in Malaysia is estimated to cost between USD 23.5 to 55. 4 million (RM 91.7 to RM 216.5 million) per year. In Germany, for example, the direct cost of severe hypoglycemia from Type II DM was estimated at USD 54,980 (€ 44,338) per 100,000 inhabitants for 1997–2000 [27,28]; in comparison, the cost estimate for Malaysia is significantly higher, amounting to at least USD 526,585 (RM 2,058,963) per 100,000 inhabitants. Although the length of stay and the unit cost of care are not necessarily higher, hypoglycemic care at hospitals has become a burden with the number of admissions required.

A significant portion of hypoglycemic episodes are treated at home without the assistance of medical services either at the A&E or hospital [29], indicating that the cost burden estimated by this study is only the tip of the iceberg. More frequent hypoglycemic events could have a significant impact on the quality of life, in addition to their indirect costs of limiting work capacity and productivity.

## Conclusions

The findings of this study show that severe hypoglycemia in patients with diabetes has a significant impact on resource utilization. Cost of hypoglycemia was found to be higher among patients with a higher severity level and in the 40–59 age group. Although a seemingly simple condition, hypoglycemia can result in substantial economic burden for the national health-care system, ranging from 0.4% to 1.0% of the annual MOH budget. Diabetic management programs should focus on prevention of hypoglycemic episodes and health education. This could minimize hypoglycemic risk and, in turn, reduce overall health spending, minimize the fear of hypoglycemia episodes, and improve compliance in diabetes management. The high cost of hypoglycemia management calls for a personalized approach to glycemic control and development of better guidelines for clinical decision making in diabetes control strategies.

## Abbreviations

DM: Diabetes Mellitus
O&G: Obstetrics and Gynaecology
ICU: Intensive Care Unit
RM: Ringgit Malaysia
USD: US Dollars
IQR: Interquartile Range
